# Mechanical and Electrical Properties of Graphene Oxide Reinforced Copper–Tungsten Composites Produced via Ball Milling of Metal Flakes

**DOI:** 10.3390/ma15217736

**Published:** 2022-11-03

**Authors:** Fei Lin, Ruoyu Xu, Mingyu Zhou, Robert J. Young, Ian A. Kinloch, Yi Ding

**Affiliations:** 1 Department of Materials, National Graphene Institute, University of Manchester, Manchester M13 9PL, UK; 2 Department of Materials, Henry Royce Institute, University of Manchester, Manchester M13 9PL, UK; 3 Department of High-End Electrical Material, Global Energy Interconnection Research Institute Europe GmbH, 10623 Berlin, Germany; 4 State Key Laboratory of Advanced Power Transmission Technology, Global Energy Interconnection Research Institute Co., Ltd., Beijing 102209, China

**Keywords:** nanomaterials, metal matrix composites, mechanical testing, electrical conductivity

## Abstract

Copper–tungsten (Cu-W) composites are widely used in high-power and -temperature electrical applications. The combination of these metals, however, leads to compromised physical and electrical properties. Herein, we produce Cu-W-graphene oxide (Cu-W-GO) composites to address this challenge. To ensure uniform density composites, the as-received metal powders were flattened into a flake morphology by ball milling and then mixed with up to 0.5 wt.% GO flakes. The green forms were processed using spark plasma sintering. The GO was found to be well-dispersed amongst the metallic phases in the final composite. The addition of GO reduced the relative density of the composites slightly (4.7% decrease in relative density at 0.5 wt% GO loading for the composites processed at 1000 °C). X-ray diffraction confirmed good phase purity and that no carbide phases were produced. GO was found to improve the mechanical properties of the Cu-W, with an optimal loading of 0.1 wt.% GO found for ultimate compression strength and strain to failure, and 0.3 wt.% optimal loading for the 0.2% offset yield strength. Significantly, the electrical conductivity increased by up to 25% with the addition of 0.1 wt.% GO but decreased with higher GO loadings.

## 1. Introduction

Copper–tungsten (Cu-W) composites are used in electrical contacts, heat sinks in high voltage power devices and arc-resistant electrodes due to their unique combination of properties. In particular, Cu-W composites possess a high hardness, relatively good electrical and thermal conductivities, a low thermal expansion coefficient, and high oxidation, erosion and arc resistances at extremely high temperatures [1]. Cu-W composites are produced typically by the high temperature infiltration of liquid Cu into a porous W skeleton. This route, however, leads to poor uniformity of the microstructure and thus to relatively low thermal and electrical conductivities due to scattering from defects and voids [2,3]. It is also difficult to fabricate a fully dense Cu-W composite using this method, leading to poor hardness and arc erosion resistance, limiting the operating life of high voltage (e.g., 200 kV) switch gear made from such materials [1]. Hence, innovative methods such as mechanochemical synthesis [4], powder injection moulding [5], mechanical alloying [6], spark plasma sintering [7], and novel additives such as graphene [8] are required to improve the physical properties of the Cu-W composites for all applications.

Graphene, with its good mechanical and electrical properties [9], is potentially an excellent reinforcement for Cu-W composites. Although progress in graphene reinforced metal matrix composites (MMCs) is far behind that of polymer matrices [10,11,12], interest in graphene-MMCs has grown rapidly over the last decade [13,14,15,16,17,18,19], with Al matrix composites [20,21,22] being the most researched. Powder metallurgy (PM) is the most widely used route for producing graphene-MMCs [21,23,24,25,26,27] with other approaches including molecular level mixing between carbon nanotubes [28] or graphene oxide and the metal ions [13,19], chemical vapour deposition of or graphene on metal foils or powders [14,18,29] and electrodeposition/electroless deposition [27]. Under the right conditions, graphene is found to enhance the properties of the MMCs. For example, Li et al. [30] reported that graphene oxide (GO) could be dispersed in the Al through a direct electrostatic interaction, to give a 18% and 17% increase in elastic modulus and hardness upon the addition of 0.3 wt.% GO. Dong et al. [31,32,33] improved the arc erosion properties, electrical conductivity and mechanical properties of 70 wt.% W-30 wt.% Cu composites by adding reduced graphene oxide (rGO). More recently, the same group has shown that precoating the graphene oxide with tungsten carbide can control the interface between the Cu and graphene [34].

In this present study we explore a powder production route for Cu-W-GO composites across a range of graphene concentrations. It is well known that there is an optimal loading of graphene to enhance physical properties for both polymer and metal matrix composites. However, previous research generally used a single graphene loading in a given study to establish whether graphene can give benefits in Cu-W composites rather exploring the role of the GO concentration in controlling the structure and properties of the composite. Importantly, ball milling was used in this present study to flatten the Cu and W flakes to ensure a good packing between the flakes and the GO in the green composite. These dispersions were then processed using spark plasma sintering at 1000 and 1100 °C, temperatures chosen to be just below and the above the melting point of the Cu. The microstructure of the resulting materials was characterised using a combination of X-ray diffraction (XRD), Raman spectroscopy and electron microscopy as a function of GO loading. The role of GO loading upon the microstructure, mechanical properties and electrical properties of the composites was then established.

## 2. Materials and Methods

### 2.1. Composite Production

**Figure 1:**
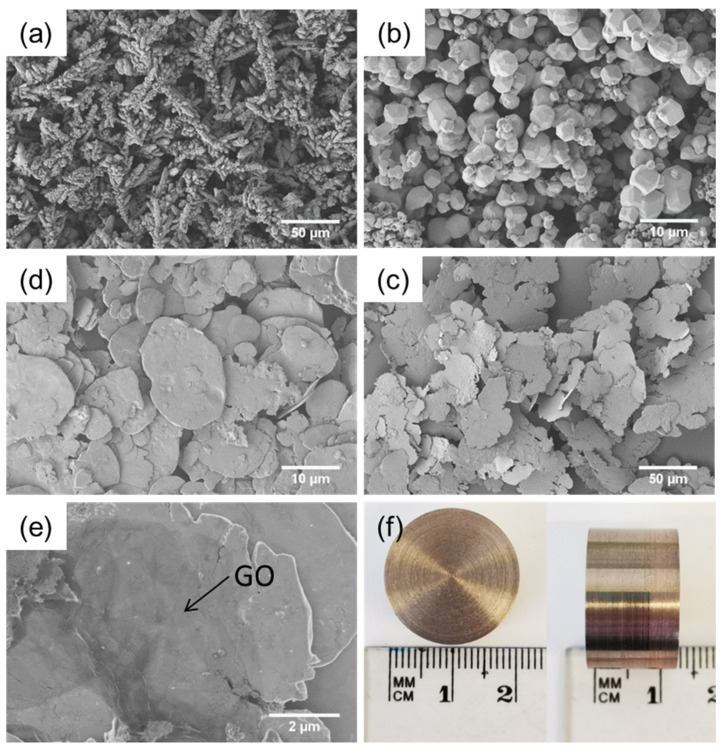
SEM images for raw (**a**) Cu, (**b**) W powders, (**c**) Cu flakes ball milled for 1 h, (**d**) W flakes ball milled for 1 h, (**e**) the flattened Cu-W powders with GO flakes (darker area), (**f**) the final bulk Cu-W-GO composite produced by SPS in the top and side view.

Cu and W powders were purchased from Beijing Youyan Powder New Materials Company. Scanning electron microscopy (SEM) demonstrated that the Cu powders comprised dendritic aggregates with a primary particle size of 1–2 µm (Figure 1a), characteristic of materials produced by the electrolysis production route [1]. The W had a polyhedral morphology (Figure 1b), indicating it was produced by the reduction of tungsten oxides [2]. The W powders had a bimodal diameter distribution, with the modes at 2.5 µm and 0.5 µm. Some nanoparticles were seen on the surface of W powders and were assumed to be tungsten oxides.

GO suspensions with a concentration of 1 mg/mL in deionised water were prepared using a modified Hummers method, as described elsewhere [35]. Electron microscopy showed that the GO was primarily monolayer with the diameters of the flakes bimodally distributed with modes at ~20 µm and ~7.5 µm. XPS showed that the C/O ratio in the GO was 2.62.

Preliminary trials found that mixing the as-received materials resulted in a poor dispersion of powder in the green composites (Figure S1). Hence, the as-received metal powders were ball-milled to flatten them so that high packing densities could be achieved during sintering (Figure 1d,c). The Cu and W powders were milled separately in a 500 mL ZrO_2_ grinding jar using 2 mm ZrO_2_ grinding balls (ball-to-powder weight ratio of 20:1) at a speed of 350 rpm (Retsch PM100) in an ethanol dispersant. A milling cycle of 5 min milling followed by 15 min rest was used to prevent the temperature rising above the boiling point of the ethanol. The powders were processed for a total milling time of an hour, which was found to be the optimal time for obtaining flat, high aspect ratio flakes. The flakes were collected and the mixed in a mass ratio of 20:80 Cu:W in ethanol and stirred for 15 min at 300 rpm. The mixed flakes were finally added to the GO suspension such that the final composites would have nominal loadings of 0, 0.1, 0.3 or 0.5 wt.% GO. This Cu-W-GO mixture was stirred for a further 30 min at 300 rpm and then dried on a hot plate.

The Cu-W-GO mixtures were consolidated by SPS (FCT HPD 25; FCT Systeme GmbH). Then, 50 g of the mixed Cu-W-GO powder was poured into a graphite mold lined with a graphite foil so that the sample could be easily removed after processing. The green pellets were heated initially from room temperature to the sintering temperature of either 1000 °C or 1100 °C. When the sintering temperature had been reached, the ram applied a pressure of 60 MPa for 5 min, after which the ram pressure was released, and the sample was cooled to room temperature. A ramp rate of 50 °C/min was used for heating and cooling. No loss of Cu was found for samples sintered at 1000 °C, whereas a little Cu was lost for the samples sintered at 1100 °C due to evaporation.

### 2.2. Characterisation

The morphology and the size of the as-received and milled metal powders were studied using scanning electron microscopy (SEM, Tescan Mira3 FEG-SEM). The relative density of the composite samples was measured by using an Ohaus analytic balance with a density determination kit based on Archimedes’ principle. Transmission electron microscopy (TEM, FEI Tecnai TF30 FEG-TEM), SEM (ZEISS Sigma FEG-SEM) and Raman spectroscopy (Renishaw inVia Raman System with 633 nm HeNe laser), X-ray photoelectron spectroscopy (XPS) were used to study the as-produced GO. These samples were prepared by depositing a drop of an aqueous solution of the as-made GO onto the relevant sample holder. SEM and Raman spectroscopy was also used to measure the morphology of polished sections of the sintered composites, including the state of dispersion of GO in the composite. X-ray diffraction (XRD, PANalytical X’Pert Pro X’Celerator X-ray diffractometer with Cu Kα radiation (λ = 0.15418 nm) was used on the polished samples to identify the phase of the sintered materials, including any potential interfacial carbides.

Nanoindentation (MTS nanoindenter XP) tests were conducted on the polished samples to determine the Young’s Modulus of the composites. For each sample, 64 points (an array of 8 × 8) were indented with a spacing of 50 μm and a depth of 1000 nm, at a strain rate of 0.05 s^−1^. Mechanical compression tests were conducted on the Cu-W-GO composites using an Instron 5569 H1549 50 kN T300 static testing machine at room temperature. Pillar samples with an aspect ratio of 1.5 (3 mm diameter, 4.5 mm height) were cut from SPS bars using electrical discharge machining with their loading axis parallel to the extrusion direction. These pillars were compressed uniaxially at a cross-head displacement rate of 0.3 mm/min until failure. The tests were performed using lubrication in order to minimise friction between the pillar and the anvils. The load–displacement data from the load frame were corrected for machine compliance prior to calculating the stress and strain.

The electrical conductivity of the samples was measured in the length direction using a four-point probe. The spacing between the probes was 1.1 mm. The test specimens were machined to around 15 mm (length) × 4 mm (width) × 2 mm (thickness) in dimensions and the accurate size of the specimens measured using a spiral micrometer. The specimens were polished with grinding paper (grit size P1200) before each test. For each GO loading, three specimens were tested.

## 3. Results and Discussion

### 3.1. Morphology of the Metal Powders following Ball Milling

A uniform dispersion of the GO within the Cu and W powders is key to obtaining good physical properties. However, it was difficult to produce the composites from the as-received metallic powders due to topological incompatibility between the matrix and the filler. Thus, wet ball milling was introduced to transform the as-received metal powders into flakes. The Cu and W powders were milled for different times (10 min, 20 min, 30 min, 40 min, 50 min, 60 min, 120 min, 180 min and 240 min) and then examined using SEM.

The morphology of the Cu powders varied with the ball milling time and is illustrated in Figures S2 and S3 at low and high magnification, respectively. The dendritic Cu powders went through the processes of deformation, cold welding, squeezing, and finally fracturing during the ball milling. The aim of the milling process in this work was to obtain flakes with the maximum possible aspect ratio before fracture occurred such that the flakes had a high, flat surface area to form an interface with the GO, whilst maintaining the good mechanical properties of the metal. SEM analysis demonstrated that 60 min ball milling was optimal and produced the largest and thinnest flakes, without fracture.

The morphology of the W powders also changed during the ball milling process (Figures S4 and S5). Similar to the Cu, the W powders gradually experienced deformation, cold welding, squeezing and fracturing steps during the ball milling process. However, due to the bimodal powder diameter distribution, the morphological transformation was not uniform within the W sample; the larger W polygonal powders flattened into flakes after only 10 min milling, whereas the smaller W powder particle were flattened much slower and finally became flakes after 40 min milling. The surface of the larger W powders became very smooth after milling for 30 min whereas the surfaces of smaller powder particles was smooth by 60 min. Above 60 min, cracks or defects started to form.

### 3.2. Microstructure of the GO

**Figure 2:**
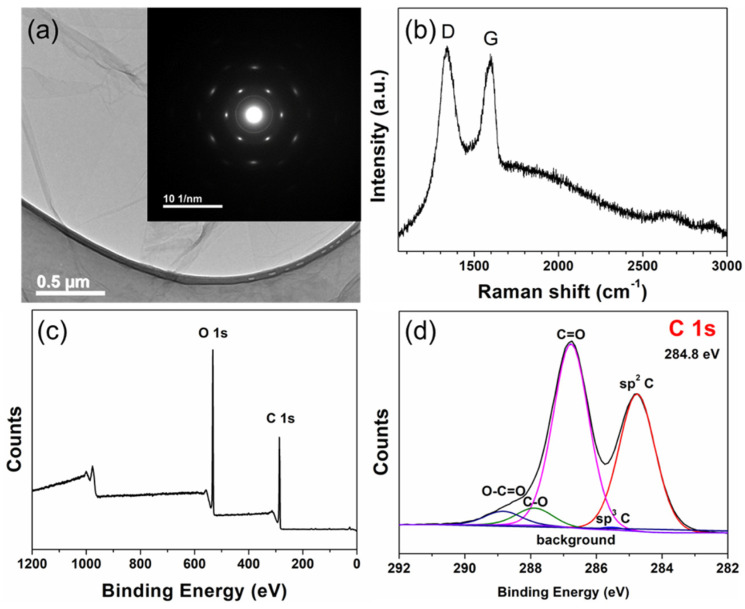
Characterization of the as-produced GO: (**a**) TEM image of GO on lacey carbon film with an inset showing a selected area electron diffraction (SAED) pattern, (**b**) Raman spectrum with the characteristic D and G bands, and (**c**) an XPS survey spectrum and (**d**) an XPS C 1s spectrum.

TEM imaging and SAED demonstrated that the GO was predominantly monolayer with some folding and wrinkling of the flakes (Figure 2a). SEM showed that the diameters of the flakes were bimodally distributed with modes at ~20 µm and ~7.5 µm. Raman spectroscopy showed the characteristic D and G bands at 1340 cm^−1^ and 1586 cm^−1^ (Figure 2b) upon a fluorescence background. The intensity of the D and G bands are approximately equal which is characteristic of as-made GO [35].

As is shown in Figure 2c, only the C 1s and O 1s peaks were identified from the survey XPS spectrum, indicating the absence of impurities. The functional groups of GO can be analysed from the C 1s spectrum (Figure 2d). Based on Cao et al. [36], C 1s was assigned to 284.8 eV, and composed of sp^2^ C, sp^3^ C, C-O, C=O, and O-C=O. Specifically, the C 1s and O 1s accounted for 72.4 at. % and 27.6 at. %, respectively to give a C/O ratio of 2.62. The atomic percentages of sp^2^ C, sp^3^ C, C-O, C=O, and O-C=O were 39.12%, 0.47%, 51.62%, 4.91%, and 3.87%, respectively.

Maintaining the structural integrity and high aspect ratio of the GO flakes is vital for mechanical reinforcement. Ball milling of Cu, W and GO together was found to result in folding and restacking of the GO. Instead, we added the GO suspensions to the powders after all the ball milling steps had been completed.

### 3.3. Microstructure of the Cu-W-GO Composites

**Figure 3:**
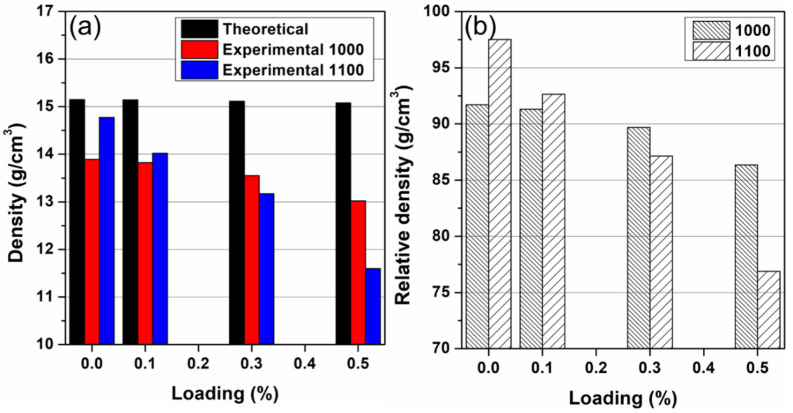
(**a**) Theoretical and experimental densities and (**b**) relative densities for the Cu-W matrix composites with different loadings of GO, processed at 1000 °C and 1100 °C.

The Cu-W-GO composite bars produced by SPS were 20 mm in diameter and 14 mm high (Figure 1f). The theoretical density for the unreinforced Cu-W is 15.15 g/cm^3^ whereas the measured density of the sample sintered at 1000 °C was 13.9 g/cm^3^, giving a relative density of 91.7% (Figure 3). The relative density decreased to 91.1%, 89.7%, and 86.4% upon the addition of 0.1, 0.3 and 0.5 wt.% graphene, respectively. The relative density for unreinforced composite at 1100 °C was significantly higher (97.5%) than that sintered at 1000 °C but the addition to the graphene led to larger decrease in the relative density, with a density of just 76.9% being obtained at 0.5 wt.% GO loading. Similar decreases in relative density have been observed in metal matrix graphene composites produced by powder metallurgy which is due to the graphene frustrating the packing and sintering of the powders, leading to the formation of pores [26]. The larger decrease in relative density that we observed at the higher sintering temperature and with higher GO loadings may be due to the GO keeping the structure more open, allowing Cu vapor to escape.

**Figure 4:**
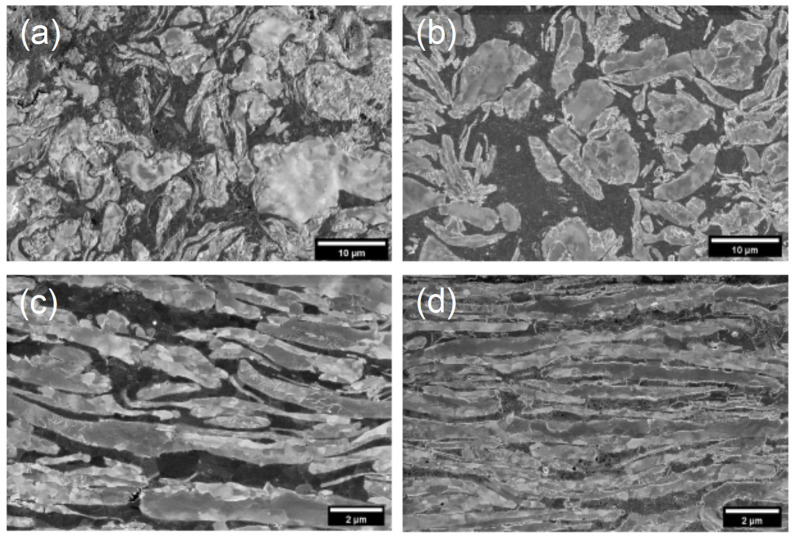
SEM micrographs for polished sections of (**a**,**c**) Cu-W and (**b**,**d**) Cu-W-0.1 wt.% GO sintered at 1000 °C viewed parallel (**a**,**b**) and perpendicular (**c**,**d**) to the extrusion direction.

SEM images and EDS mappings of the Cu-W and Cu-W-0.1 wt.% GO composites sintered at 1000 °C are shown in Figure 4 and Figures S6–S10, respectively. It can be seen that the initial flake morphology was preserved during sintering, justifying the ball milling processed used to flatten the powders prior to use. The phases are aligned perpendicular to the direction of loading during the sintering processes. Furthermore, it was found that the Cu and W were uniformly dispersed in the bulk composites with the Cu dispersed at the grain boundaries of W and vice versa.

The EDS results for the Cu-W and Cu-W-0.5 wt.% GO sintered at 1000 °C and 1100 °C are shown in Figures S6–S10, including the SEM, the combined image, and the elemental maps for Cu, W, O and C, and the integrated spectrum for the whole mapping area. Notably, the C element was mapped for comparison with the composites with GO incorporated. In general, the Cu and W phases were uniformly uniformly dispersedin the Cu-W sample, with a good Cu continuous network. This was to the result of the Cu being able to flow around the W during the processing at high temperature, leading to interconnection of the phases. A few oxide particles, which probably formed during the high temperature sintering process, are located at the grain boundaries of the W. The composition of the mapping area was 77.3 wt.% W, 21.9 wt.% Cu, and 0.8 wt.% C, a little lower than the nominal of Cu-W 20/80 ratio.

In comparison, more oxide particles were present in the Cu-W-0.5 wt.% GO composites. GO rather than graphene was used to produce a good slurry and hence a better distribution in the green pieces. However, GO is highly oxidative at high temperature, hence as it was reduced during heating, it would have oxidised the metals. In addition, dispersion of the phases in the 0.5 wt.% sample was less homogeneous than the Cu-W composite; the continuity of the Cu in the composite is interrupted by the pores and the oxides. The elemental composition of the composites was 63.8 wt.% W, 32.3 wt.% Cu, 2.1 wt.% C and 1.8 wt.% O. The percentage ratio of Cu-W is a little higher than the initial 20/80 and relative high oxygen content is believed to be from the tungsten oxides as well as the GO.

The elemental distribution and microstructure for composites processed at 1100 °C is very similar to the composites sintered at 1000 °C. A small difference is that the agglomeration is more severe for composites at 1100 °C than that at 1000 °C, especially for the composite with higher loadings of GO such as 0.5 wt.%.

**Figure 5:**
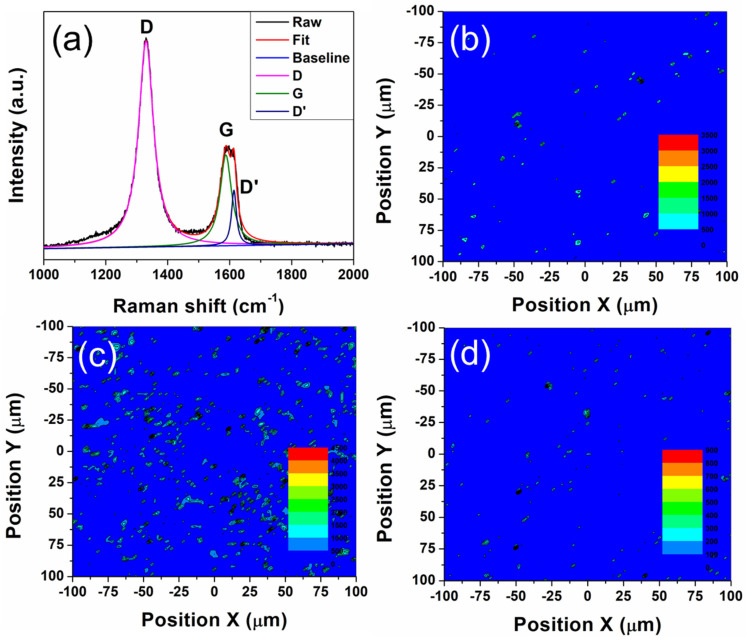
(**a**) Raman spectrum for a Cu-W composite with GO, including the raw spectrum and the curves for the fit spectrum, the baseline, D, G and D’ spectra. Raman mapping of the D band for Cu-W matrix composite with (**b**) 0.3 and (**c**) 0.5 wt.% GO sintered at 1000 °C. Raman mapping (**d**) for Cu-W matrix composite with 0.3 wt.% GO sintered at 1100 °C.

A good dispersion of the GO (at this stage reduced) in the final sintered Cu-W composite is key in obtaining good physical properties. It is difficult to distinguish GO from the matrix using either optical or electron microscopy. Thus, Raman spectroscopy was used to confirm the existence and the dispersion of GO in the composite [21,37,38]. The Raman spectrum of GO in the bulk composite after consolidation is shown in Figure 5a (c.f. Figure 2b for the spectra from the as-received materials). The spectrum was fitted by 3 Lorentzian type curves, D, G and D’, with positions at 1330 cm^−1^, 1587 cm^−1^ and 1614 cm^−1^ Raman shift, respectively. The D band results from a disordered structure within the graphene [39], hence Raman spectroscopy is very useful for characterising the disordered structure in sp^2^ hybridised carbon materials such as graphene [36]. The G band is from the stretching of the C-C bond in graphene materials and is common to all sp^2^ hybridised carbon materials. The G band can, however, split into two bands, G band and D’ band, when defects are randomly distributed in the graphene. This splitting is mainly due to the interaction between the localised vibrational modes of the defects and the extended phonon modes of graphene. The D/G band intensity ratio of the processed GO is about 2.28, much higher than that of the as-produced material (Figure 2b), indicating that composite sintering process introduced defects into the GO [39]. A similar increase in the D/G ratio has been reported upon the ball milling of GO thought also to be due to the introduction of defects [40]. Considering the complicated nature of the G band region, the D band was used as a reference for the Raman mapping. The Raman mapping results are shown in Figure 5b–d, displaying the dispersion of GO in the composites sintered under 1000 °C and 1100 °C, respectively. These maps confirm that the GO uniformly dispersed in the Cu-W matrix composites.

### 3.4. Phase Purity

**Figure 6:**
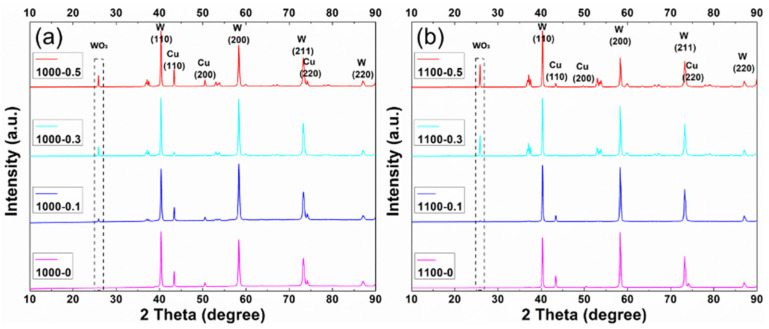
XRD spectra for Cu-W matrix composites with different loadings of GO, sintered at (**a**) 1000 °C and (**b**) 1100 °C. The labelled peaks are from W and Cu, and the other unlabelled peaks are from WO_3_, with the main reflection from WO_3_ is highlighted to the dotted boxes. Figure S11 gives the reference X-ray reflections for Cu, W and WO_3_.

XRD patterns for the composites sintered at 1000 °C and 1100 °C are shown in Figure 6, in the 2θ range between 10° and 90°. In this region, some main Bragg reflections from W and Cu are present and labelled in the plots, including W (110), W (200), W (211), W (220), Cu (111), Cu (200), and Cu (220). There are also some reflections from WO_3_. The relative peak intensity of Cu is lower at 1100 °C than that at 1000 °C, which is in agreement with the partial loss of Cu when processed at 1100 °C. Finally, the relative peak intensity for WO_3_ increases with higher loadings of GO and higher processing temperatures, due to the scavenging of the oxygen from the GO.

### 3.5. Mechanical Properties

**Figure 7:**
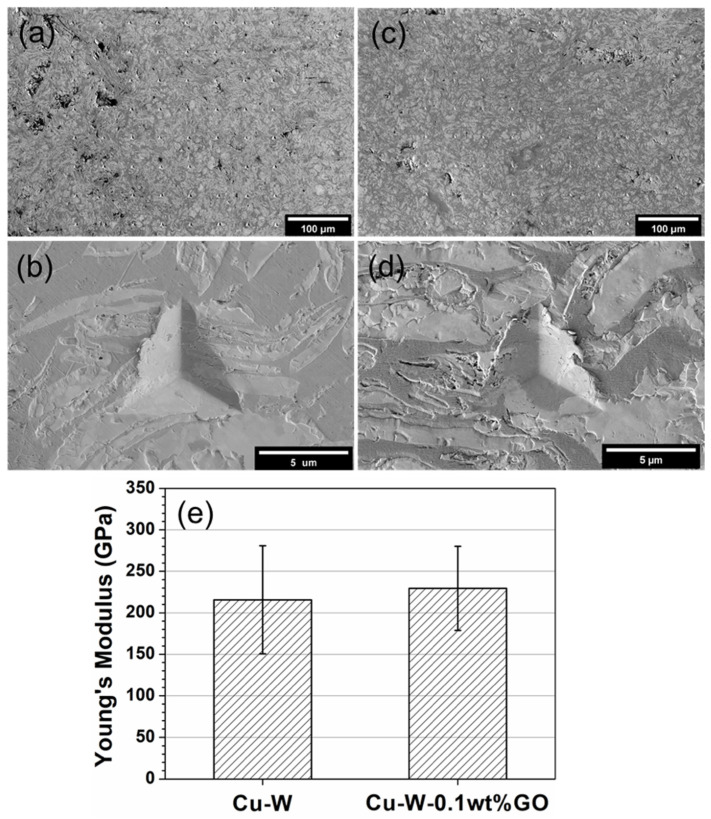
SEM image for the (**a**) Cu-W sample showing the whole indented area, and (**b**) one selected indent; SEM image for the (**c**) Cu-W-0.1 wt.% GO sample showing the whole indented area, and (**d**) one selected indent. (**e**) Values of the Young’s moduli of the Cu-W and Cu-W-0.1 wt.% GO materials.

The Young’s modulus the materials was determined using nanoindentation. SEM rather than optical microscopy was used to evaluate the results of the nanoindentation tests due to small indents being produced from such a stiff material. The 8 x 8 arrays from the tests and higher magnification micrographs of typical indents can be seen in Figure 7. The indents that were found to be near defects were excluded from the analysis. To summarise the properties derived from the two samples examined (Cu-W and Cu-W-0.1 wt.% GO), the Young’s modulus results obtained from the nanoindentation tests are also shown in Figure 7. The mean value of the Young’s modulus of the Cu-W is 218 ± 89 GPa, which increases to 229 ± 55 GPa for the Cu-W-0.1 wt% GO. These values compare will those reported in the literature. For example, modulus values of between 160–190 GPa have been reported for a 25:75 Cu:W composite processed in the temperature range 1050–1150 °C [41]. The modulus appears to increase slightly upon the addition of 0.1 wt.% GO but this may not be significant when the errors are considered.

**Figure 8:**
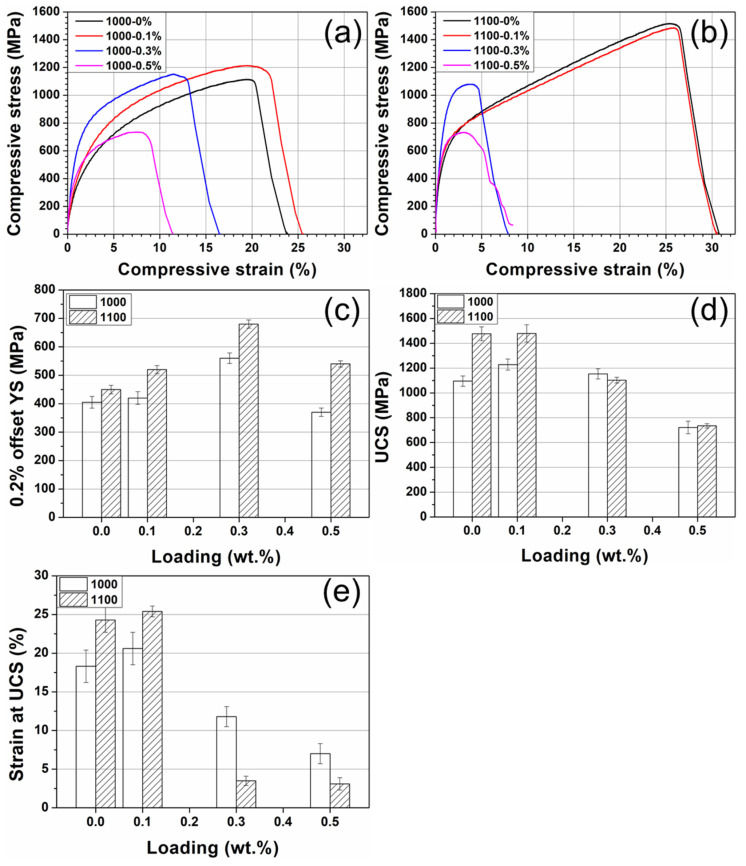
(**a**,**b**) Compressive stress–strain curves for samples with loadings of 0, 0.1, 0.3, and 0.5 wt.% GO at 1000 and 1100 °C, respectively. Each curve in the plot is the most representative one for each sample. The pillars tested had a diameter of 1.5 mm and height of 4.5 mm and 8 specimens were tested for each composition. (**c**) 0.2% offset yield strength, (**d**) Ultimate compressive stress (UCS) and (**e**) strain at UCS for Cu-W matrix composites with different loadings of GO produced by SPS at 1000 and 1100 °C.

Representative compressive stress–strain curves for all eight different production and sintering conditions are shown in Figure 8. Since the curve in the elastic region is not quite linear a 0.2% offset yield strength (YS) was used. The mean ultimate compressive stress (UCS) for Cu-W sintered at 1000 °C is 1095 ± 42 MPa, with a corresponding mean strain at failure of 18.3 ± 2.1%. Upon the addition of 0.1 wt.% GO, the compressive strength of the composite increased to 1228 ± 45 MPa and the strain to failure increased to 20.6%. However, at higher GO loadings the UCS and strain to failure dropped, with the GO embrittling the metal matrix composite. The GO did, though, improve the 0.2% offset yield strength compared to that of the control composite at all loadings studied for a 1000 °C processing temperature. However, this improvement in 0.2% offset yield strength was optimal for 0.3 wt.% GO, where it increased by 38% to 560 MPa.

The processing temperature had a significant role in the mechanical performance of the composites. Both Cu-W and Cu-W-0.1 wt.% GO processed at 1100 °C showed mechanical properties superior to all samples processed at 1000 °C. For example, Cu-W sample processed at 1000 °C possessed an ultimate compressive stress of 1095 MPa and a strain to failure of 18.3%, whereas the Cu-W sample sintered at 1100 °C had values of 1477 MPa and 24.3%, respectively. The values of UCS compare well with those reported in the literature for Cu-W composites of a similar composition [42]. However, there was significant embrittlement with a smaller plastic region for composites with 0.3 and 0.5 wt.% GO at 1100 °C with the degree of embrittlement increasing with increasing temperature. Interestingly, the Cu-W-0.3 wt.% GO samples had the optimal yield stress, regardless of the sintering temperature used. For the 1100 °C sintered sample, the 0.2% offset yield strength increases to 680 MPa which more than a 50% increase compared with the Cu-W sample.

### 3.6. Electrical Properties

**Figure 9:**
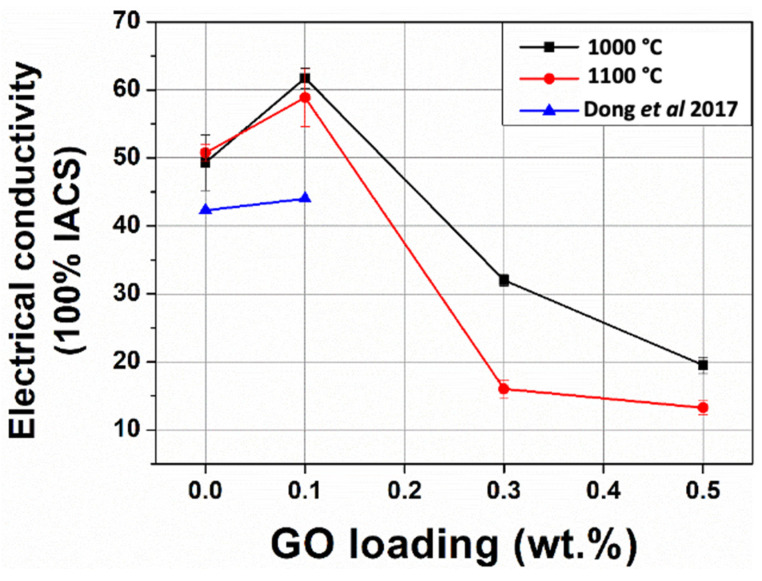
The measured electrical conductivity of samples as function of GO loading and sintering temperature. Additionally, shown are the conductivities reported in [8].

A four-point probe measurement was used to measure the electrical conductivity of the Cu-W-GO composites (Figure 9). The spacing for each probe was 1.1 mm, which is much larger than the length of the 1000 °C Cu-W phase domains, meaning the results should be typical for the whole composite. The electrical conductivity for composites with 0.1 wt.% GO was higher than the unreinforced sample, with improvements of 25% and 16% at sintering temperatures of 1000 and 1100 °C, respectively. However, the conductivity for composites with 0.3 wt.% and 0.5 wt.% was significantly lower. The effects of GO loading for the electrical conductivity are similar to the ultimate compressive stress and strain. As revealed by EDS and XRD previously, more oxides (WO_3_) are formed at higher loadings, which have much lower electrical conductivity than Cu. In addition, the Cu network may be disrupted by these oxides. Finally, more porosity is found (from both relative density and SEM results) in the composites with higher GO loadings, making electron flow less efficient in the composites.

The electrical conductivity results for the Cu-W matrix composites are also compared to the conductivity from Dong et al. [8] in Figure 9. As can be seen, that the conductivity of the composites produced in this work with and without GO is higher than that in their work, possibly due to the ball milling route used allowing a better Cu pathway to be obtained. Further improvements in performance might be obtained through the use of metal-doped reduced GO as demonstrated in a recent study upon arc-erosion resistance in similar materials [43].

## 4. Conclusions

We have demonstrated that flake powder metallurgy can be successfully employed to produce Cu-W matrix composites with different loadings of GO (0.1, 0.3, 0.5 wt.%) and at different sintering temperature (1000, 1100 °C). The wet ball milling of the as-received flakes was found to flatten them without causing significant fracture, overcoming the morphology incompatibility between the as-received metal powders and GO flakes. This procedure enabled Cu-W composites with densities of 91.7% and 97.5% to be obtained at 1000 and 1100 °C, respectively, with the latter higher due to the Cu being molten during processing. The addition of the GO reduced the density, due to frustrated packing and the introduction of oxides. However, the density of the 1000 °C sintered Cu-W composite dropped by only 5.35% at a 0.5 wt.% GO loading, while the decrease was much higher (20.6%) for the composite sintered at 1100 °C. X-ray diffraction and EDS showed good phase purity, with the content of undesirable oxides increasing with GO loading and sintering temperature. Raman spectroscopy confirmed that the GO was reduced but had survived the sintering process and a good dispersion was achieved even at loadings up to 0.5 wt.% GO. Compression testing found that the composites sintered at 1100 °C has better mechanical properties compared with the composite sintered at 1000 °C due to the higher density at low levels of GO loading. GO was found to improve the mechanical properties, with an optimal loading of 0.1 wt.% loading found for ultimate compressive stress (UCS) and strain to failure and 0.3 wt.% optimal loading for the 0.2% offset yield strength. For higher GO loadings (>0.3 wt%) the strength of the composite was reduced due to an increase in porosity. The electrical conductivity increased with the addition of 0.1 wt.% GO but then decreased at higher loadings. Overall, 0.1 wt.% GO sintered at 1100 °C is considered to be the best composition since the conductivity, yield strength, UCS and strain at the UCS are all improved.

## Data Availability

The authors confirm that the data supporting the findings of this study are available within the article and its supplementary material.

## Supplementary Materials

The following supporting information can be downloaded at: https://www.mdpi.com/article/10.3390/ma15217736/s1, Figure S1. SEM of the green composite formed by mixing the as-received materials mixed in a ratio of Cu20W80-0.1 wt.% GO. Figure S2. SEM micrographs for Cu powders after ball milling for 10, 20, 30, 40, 50, 60, 120, 180 and 240 min.; Figure S3. SEM micrographs at high magnification for Cu powders after ball milling for 10, 20, 30, 40, 50, 60, 120, 180 and 240 min.; Figure S4. SEM micrographs for W powders after ball milling for 10, 20, 30, 40, 50, 60, 120, 180 and 240 min.; Figure S5. SEM micrographs at high magnification for W powders after ball milling for 10, 20, 30, 40, 50, 60, 120, 180 and 240 min.; Figure S6. EDS mapping for Cu-W flake powders with 0.1 wt.% GO: SEM image, SEM image combined with elemental mapping, elemental mapping for Cu, W, C and O.; Figure S7. EDS results for Cu-W sintered at 1000 °C, including a secondary electron image, EDS layered image, elemental maps for W, Cu, O, and C, EDS map sum spectrum.; Figure S8. EDS results for Cu-W with 0.5 wt.% GO sintered at 1000 °C, including a secondary electron image, EDS layered image, elemental maps for W, Cu, O, and C, EDS map sum spectrum.; Figure S9. EDS results for Cu-W sintered at 1100 °C, including a secondary electron image, EDS layered image, elemental maps for W, Cu, O, and C, EDS map sum spectrum.; Figure S10. EDS results for Cu-W with 0.5 wt.% GO sintered at 1100 °C, including a secondary electron image, EDS layered image, elemental maps for W, Cu, O, and C, EDS map sum spectrum; Figure S11. X-ray diffraction reference data for the Cu, W and WO_3._
