# Supplementary material for: Mechanical and Electrical Properties of Graphene Oxide Reinforced Copper–Tungsten Composites Produced via Ball Milling of Metal Flakes
Source: Materials (Basel). 2022 Nov 3;15(21):7736. doi: 10.3390/ma15217736 (PMC9655810; doi:10.3390/ma15217736)
Supplement: Supplementary file 1 [file materials-15-07736-s001.zip › materials-1987022-supplementary.pdf]

Supplementary data

Mechanical and Electrical Properties of Graphene Oxide Reinforced Copper-Tungsten Composites Produced via Ball Milling of Metal Flakes

Fei Lin[1], Ruoyu Xu[2], Mingyu Zhou[2], Robert J. Young[1], Ian A. Kinloch[1*], Yi Ding[3]

[1] *Department of Materials and the National Graphene Institute, University of Manchester, Manchester M13 9PL, UK*

[2] *Department of High-End Electrical Material, Global Energy Interconnection Research Institute Europe GmbH, Berlin 10623, Germany*

[3] *State Key Laboratory of Advanced Power Transmission Technology, Global Energy Interconnection Research Institute Co., Ltd. Beijing 102209, China*

SEM of the green composite formed by mixing the as-received Cu, W and GO powders.

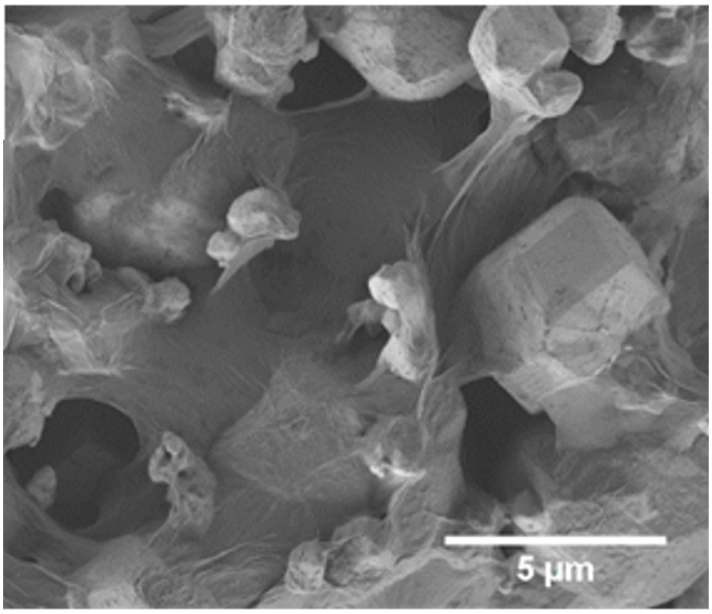

Figure S1. SEM of the green composite formed by mixing the as-received materials mixed in a ratio of Cu20W80-0.1wt.%GO.

SEM of the ball-milled of the as-received Cu and W powders

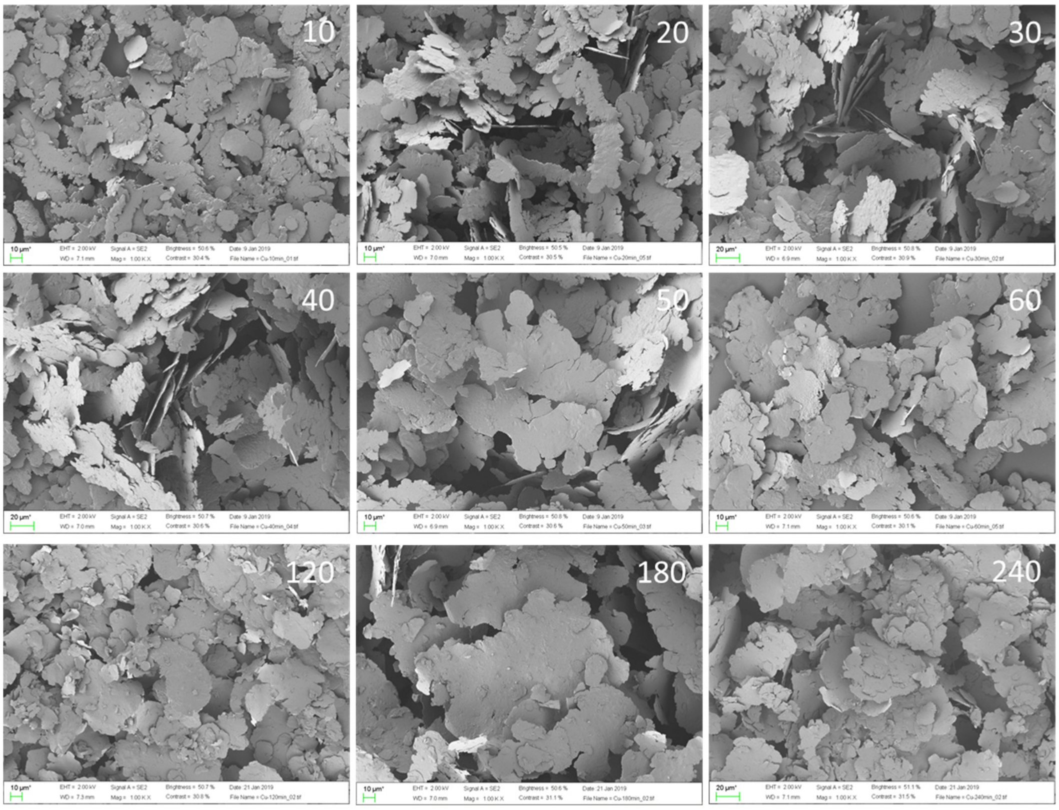

Figure S2. SEM micrographs for Cu powders after ball milling for 10, 20, 30, 40, 50, 60, 120, 180 and 240 min. Please note that the image for the as-received powder is shown in Figure 1.

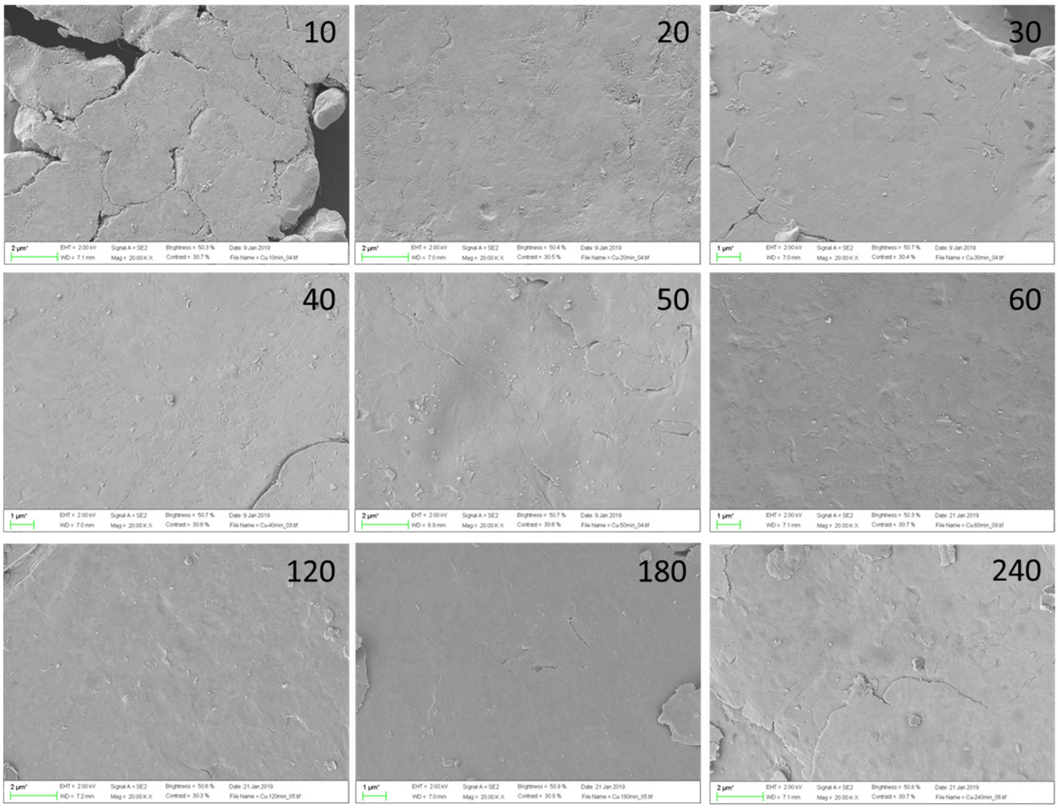

Figure S3. SEM micrographs at high magnification for Cu powders after ball milling for 10, 20, 30, 40, 50, 60, 120, 180 and 240 min.

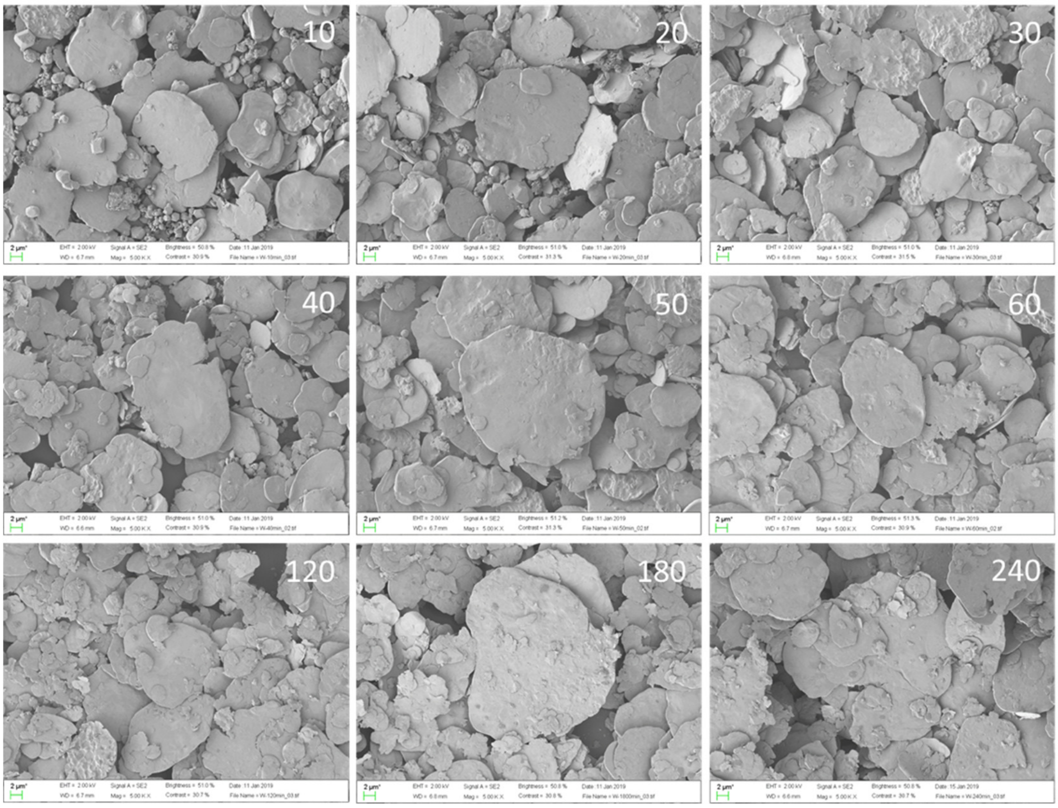

Figure S4. SEM micrographs for W powders after ball milling for 10, 20, 30, 40, 50, 60, 120, 180 and 240 min. Please note that the image for the as-received powder is shown in Figure 1.

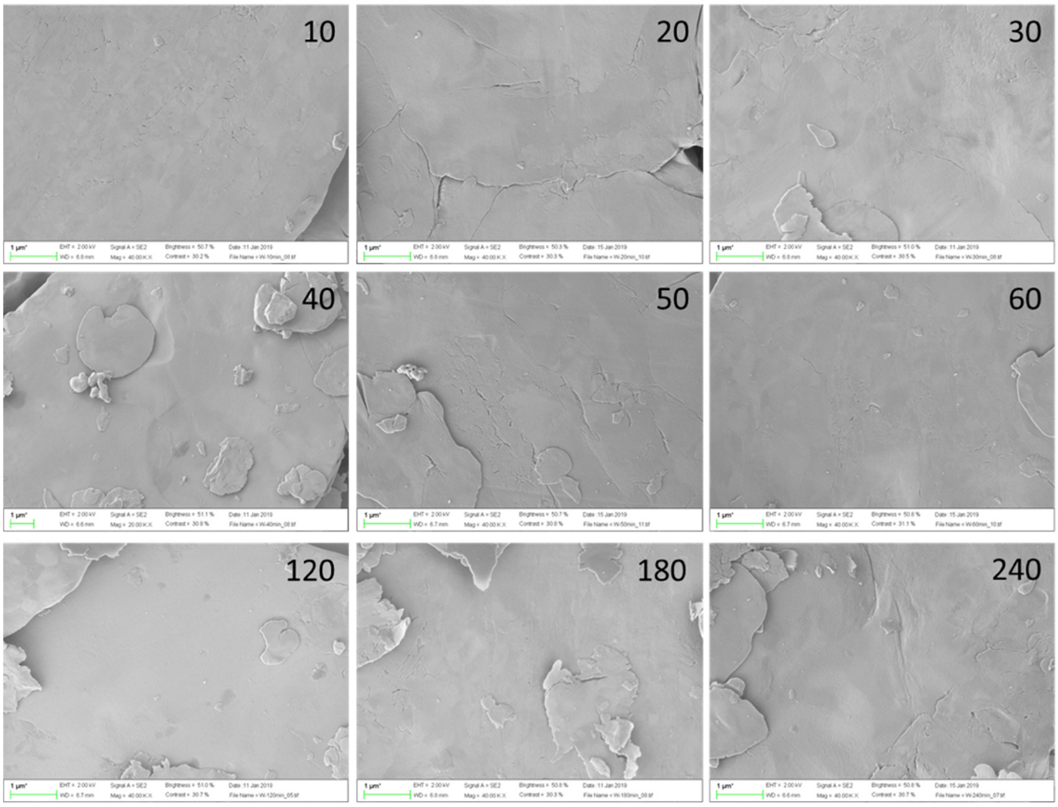

Figure S5. SEM micrographs at high magnification for W powders after ball milling for 10, 20, 30, 40, 50, 60, 120, 180 and 240 min.

Elemental mapping of the composites using EDS

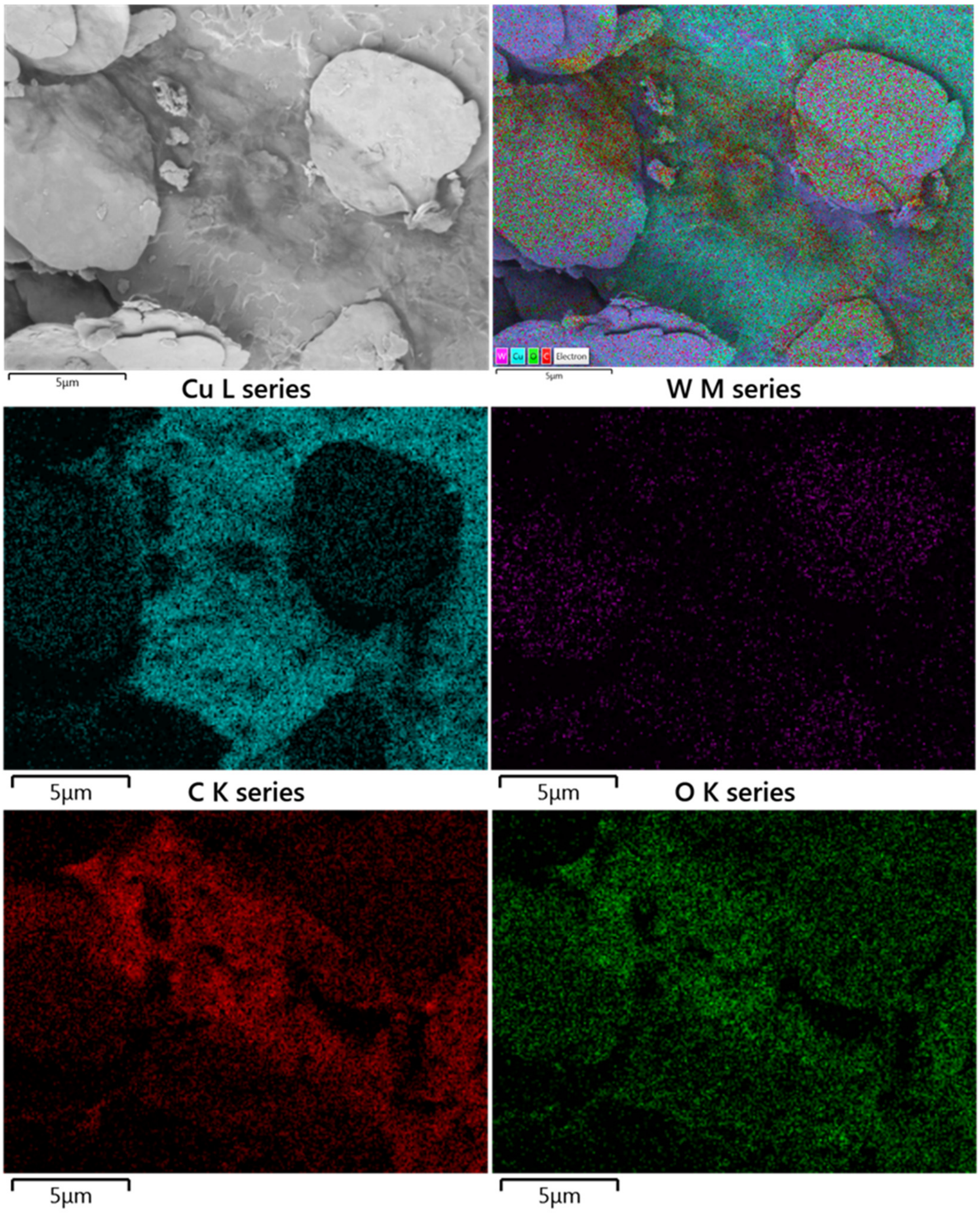

Figure S6. EDS mapping for Cu-W flake powders with 0.1wt.% GO: SEM image, SEM image combined with elemental mapping, elemental mapping for Cu, W, C and O.

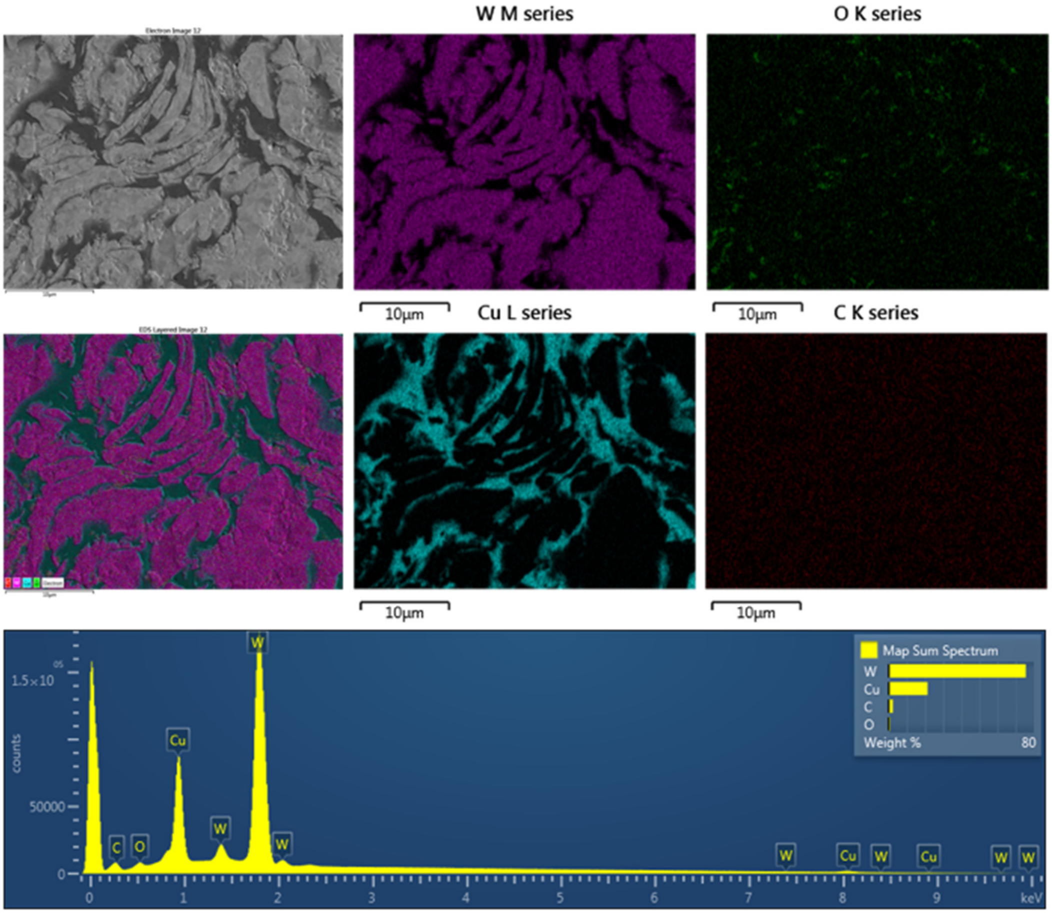

Figure S7. EDS results for Cu-W sintered at 1000 ºC, including a secondary electron image, EDS layered image, elemental maps for W, Cu, O, and C, EDS map sum spectrum.

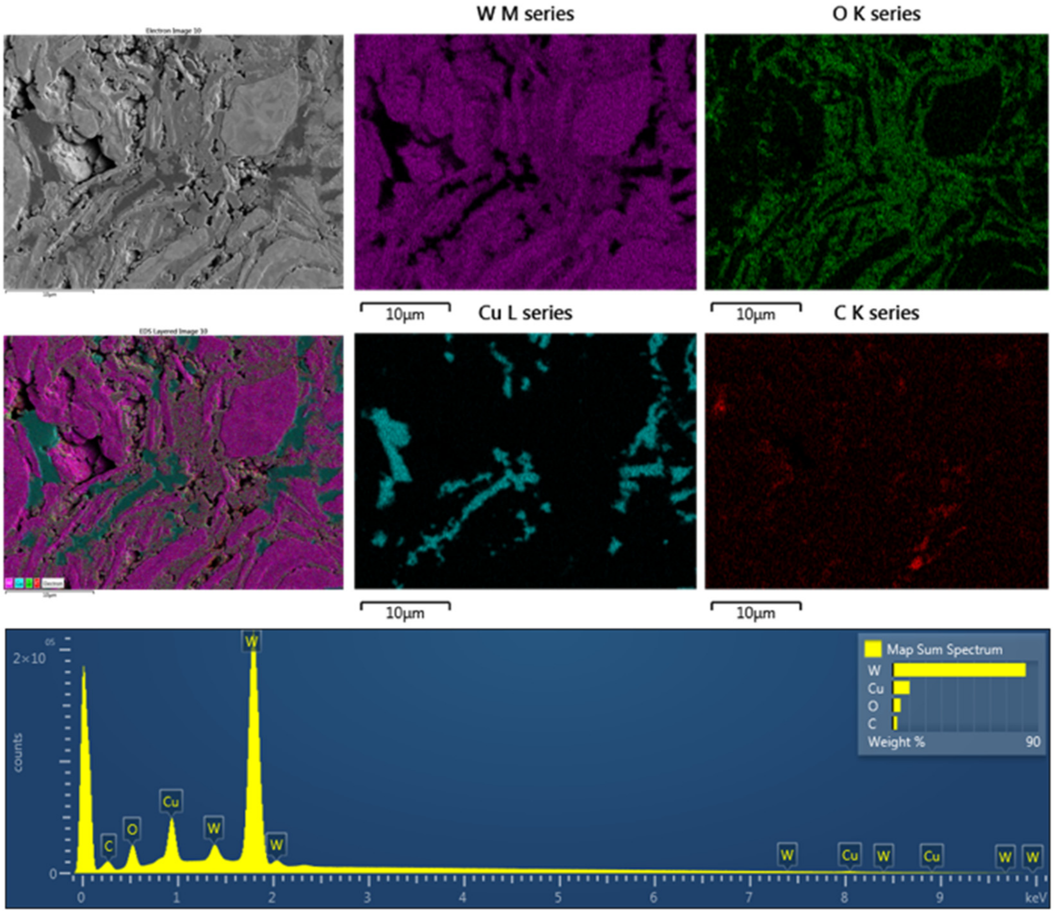

Figure S8. EDS results for Cu-W with 0.5 wt.% GO sintered at 1000 ºC, including a secondary electron image, EDS layered image, elemental maps for W, Cu, O, and C, EDS map sum spectrum.

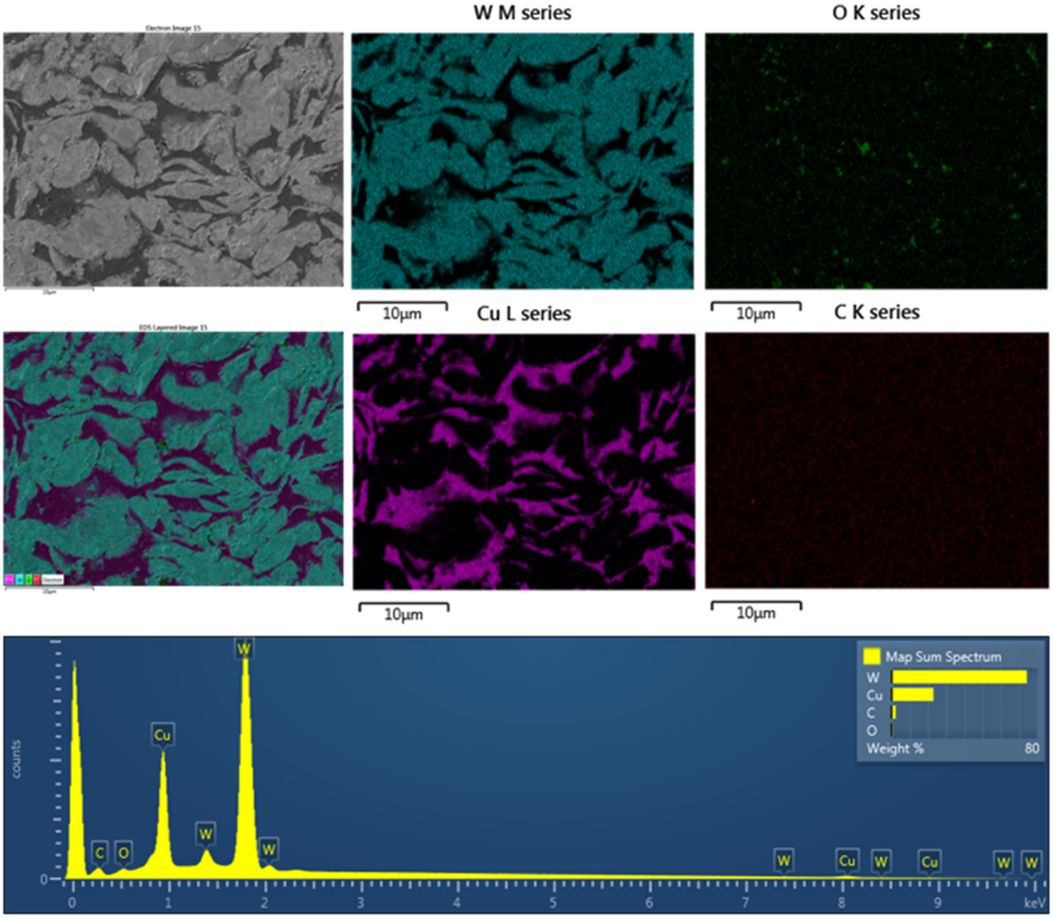

Figure S9. EDS results for Cu-W sintered at 1100 ºC, including a secondary electron image, EDS layered image, elemental maps for W, Cu, O, and C, EDS map sum spectrum.

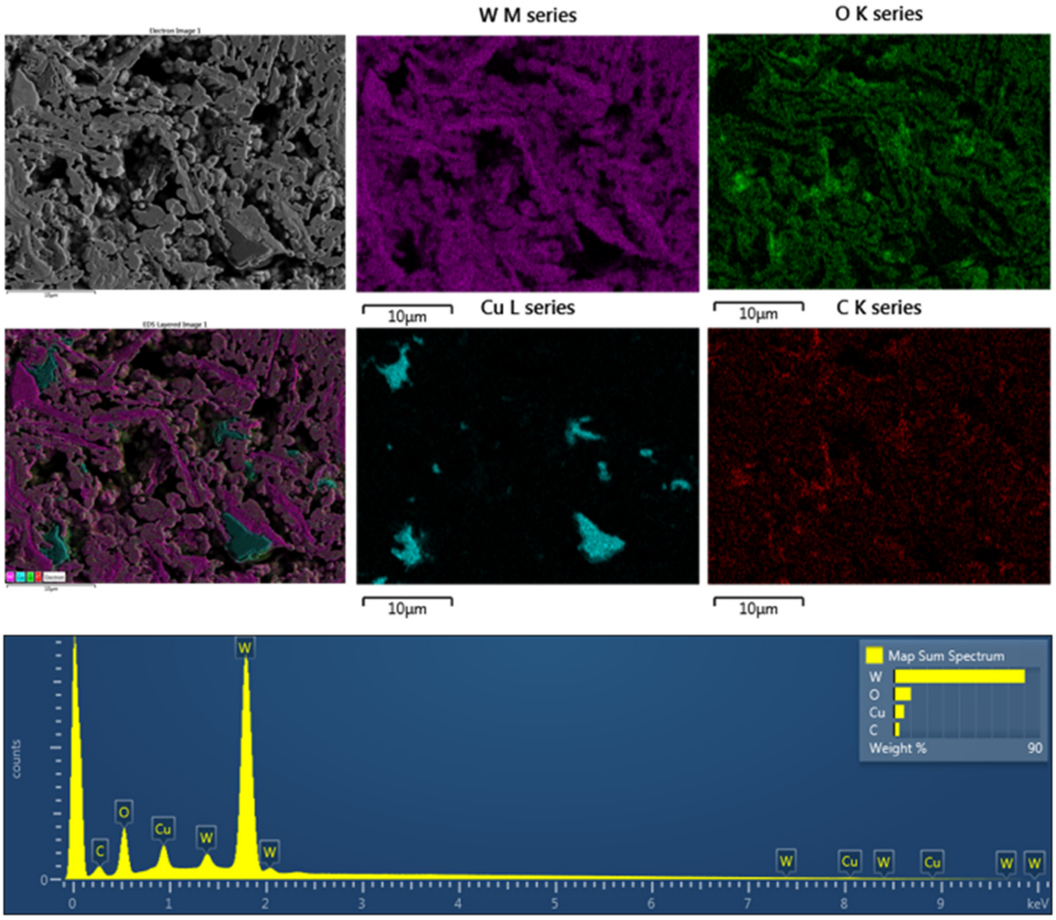

Figure S10. EDS results for Cu-W with 0.5 wt.% GO sintered at 1100 ºC, including a secondary electron image, EDS layered image, elemental maps for W, Cu, O, and C, EDS map sum spectrum.

Reference X-Ray Diffraction Patterns

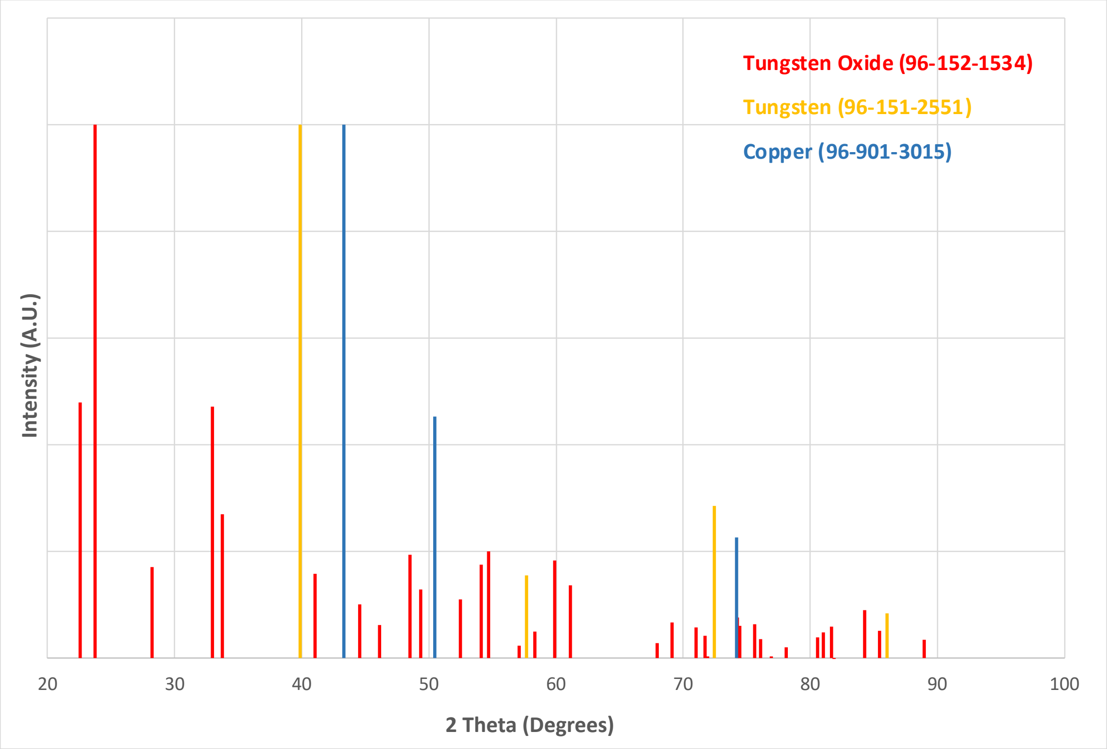

Figure S11. X-ray diffraction reference data for the Cu, W and WO_3 with their reference numbers given in the legend.
